# The Tyranny of Distance: How Hospital Transfer Affects Time to Surgery for Hip Fracture Patients

**DOI:** 10.7759/cureus.22662

**Published:** 2022-02-27

**Authors:** Melanie A Marley, Anton Lambers, Ian Marley, Lisa Welthy, Hannah Seymour

**Affiliations:** 1 Orthopaedics and Rehabilitation, Fiona Stanley Hospital, Perth, AUS

**Keywords:** orthopedic surgery, orthogeriatric medicine, time to surgery, rural health, proximal femur fracture

## Abstract

Introduction

In Western Australia, vast distances between hospitals can limit a patient’s access to timely surgical intervention. The aim was to examine the effect of patient location on outcomes.

Methods

Hip fracture data from all operative cases at the major Western Australian hospitals between 2015 and 2019 was retrospectively reviewed. A total of 5691 patients were separated into three groups based on hospital of first presentation - directly to the operative hospital (metropolitan), a hospital less than 2.5 hours by road from the operative centre (outer-metropolitan), or requiring transfer by air (rural). Impact of location on time to surgery, length of stay and 30-day and 120-day mortality was analysed.

Results

The mean time to surgery was 26.7 hours for metropolitan patients, 37.0 hours for outer-metropolitan, and 42.6 hours for rural patients. Outer-metropolitan patients were less likely to reach surgery within 48 hours than metropolitan patients (80.2% vs 91.5%, p<0.001), with even lower rates for rural patients (66.8%, p<0.001). Acute length of stay was longer for rural patients compared to outer-metropolitan (7.2 vs 5.8 days) and metropolitan patients (5.5 days) (p<0.001). There was no significant difference in 30-day or 120-day mortality for outer-metropolitan or rural patients compared to metropolitan patients despite requiring transfer. However, when considered as a whole group there was an increased 120-day mortality with increased time to surgery. Overall mortality was 8.7% at 30 days and 17.3% at 120 days.

Conclusion

Patients presenting outside the metropolitan area with a hip fracture have a longer time to surgery and longer length of stay. Delay for outer-metropolitan patients is disproportionately longer than transit time alone and may provide opportunities for improvement.

## Introduction

Hip fractures present a significant burden to the healthcare system. In the 2015-16 financial year there were 18,700 hip fractures in Australia [1]. Evidence-based guidelines for hip fractures recommend surgery within 24-48 hours [2-5], with time to surgery being an established factor affecting postoperative outcomes for these patients [3,4,6-8]. There is also a financial impact of delayed surgery with the average orthopaedic bed costing $AU1130 per day [9].

The state of Western Australia (WA) covers a very large land area at 2.6 million square kilometres, measuring more than 2,500km from the northernmost to southernmost points. Patients who suffer a hip fracture outside of the capital city of Perth usually require transfer for management at one of the three tertiary operative centres in the metropolitan region. The vast distances between hospitals in WA can limit a patient’s access to timely surgical intervention. There have been no large-scale or long-term observational studies reviewing time to surgery based on regions within WA. One smaller study looking at a single institution found a 12-hour delay to surgery in patients that required an inter-hospital transfer which was associated with increased mortality [10].

At the time of this study, WA had set a quality measure of surgery within 36 hours of presentation at the operative hospital. The Australian and New Zealand Hip Fracture Quality Care Standard set an expectation of surgery within 48 hours of first presentation to hospital. The Australian & New Zealand Hip Fracture Registry (ANZHFR) produces an annual report, which includes time to surgery. The 2016 and 2017 reports looked at all patients as one cohort with a mean time to surgery of 41 and 54 hours, respectively [11,12]. They commented on the increased time in the 2017 report as a reflection on more hospitals participating in the registry [12]. For the 2018, 2019 and 2020 reports, the time to surgery was separated into two groups, patients who were transferred and patients who presented directly to the operative hospital [13-15]. They report a mean time to surgery of 54, 53 and 47 hours for transferred patients in 2018, 2019 and 2020 reports respectively, and a mean time to surgery of 37, 37 and 36 hours for non-transferred patients in 2018, 2019 and 2020, respectively [13-15].

Increases in time to surgery for patients requiring transfer have been demonstrated at different centres previously, though in less geographically dispersed regions. A study in another Australian state showed a significantly greater time to surgery for transferred patients at 58 hours versus 41 hours for direct presentation [16]. In their study, 35% of transferred patients had surgery within 36 hours [16]. State-wide data from New South Wales from 2001-2011 found that 25% of patients underwent surgery on the day of admission, 64% by the second day of admission [17]. Patients requiring inter-hospital transfer had a significant increase in time to surgery [17] but exact increase was not reported. Data from Ontario, Canada found that transferred patients had a median time to surgery of 93 hours compared to 44 hours for non-transfer patients [18].

The primary outcome of this study was time to surgery for hip fracture patients in Western Australia based on geographical location. The hypothesis was that patients living further from the operative hospital would have a longer time to surgery than those transferred shorter distances by road or those who present directly to the operative hospital. Secondary outcome measures were length of stay and 30-day and 120-day mortality. In doing this, the study would also provide an update on hip fracture care in Western Australia and identify how we are comparing to the Australian Safety and Quality Commission Clinical Care standards by comparing WA to the ANZHFR report.

This article was previously presented as a meeting abstract at the 2020 Australian Orthopaedic Association (Western Australia) Annual Scientific Meeting on October 31, 2020.

## Materials and methods

The hip fracture database of Western Australia, the Quality of Care Registry - Hip Fractures (QoCR database), was accessed for data collection. QoCR was developed in 2011 and has been utilised across WA for the purpose of collecting hip fracture data for quality improvement. All patients with a hip fracture in WA are recorded in the database by a hip fracture nurse at participating sites. QoCR is linked to several electronic systems, which automatically feed data including ED presentation time, surgical date and time into QoCR. Other data is collected during the admission using the medical record. Data points recorded include, but are not limited to, patient name, age, gender, transferring hospital, hospital of operative treatment, ASA, fracture type, pre-admission mobility, pre-operative cognition, assessment by geriatrician, pain assessment, time of surgery, post-operative mobility, post-operative assessments (delirium, malnutrition, bone health), length of acute admission, transfer to rehabilitation, 30- and 120-day mortality. Data from the QoCR database is then uploaded to a national hip fracture registry (ANZHFR) if the site has obtained ethics approval to do so, which has progressively increased for WA hospitals since 2015. All patients since the inception of the QoCR database were retrospectively reviewed over a five-year period from January 1st, 2015 to December 31st, 2019.

Patients were separated into three groups based on hospital of first presentation - those presenting directly to the site of their definitive procedure in Perth (metropolitan), those presenting to a hospital less than 2.5 hours by road to the operative centre (outer-metropolitan), and those presenting to a hospital more than 2.5 hours by road which typically requires transfer by air (rural).

There were 6672 patients in the initial dataset. Patients were excluded from the study if no surgical procedure was performed, or if time to surgery data was missing, which excluded 815 patients. Patients were also excluded if they were not treated at a tertiary site, excluding a further 137 patients. Finally, 29 patients were excluded if their mode of transfer (road vs. air) was unclear. Western Australian Country Health Service (WACHS) allows country hospital sites to determine their own policies on transfer based on both the site and patient presentation, with a guide cut-off for road travel of 200km - approximately 2.5 hours [19]. If mode of transport remained unclear from the data points gathered in the QoCR database, that patient was excluded as they could not be allocated into our outer-metropolitan or rural group. There were a final total of 5691 patients included in the analysis. Following exclusions, patients were then separated into the three groups outlined above. Time to surgery was calculated from presentation to theatre arrival time. For hospital presentations, this was calculated from the time of arrival at the Emergency Department of the first hospital. For patients retrieved from a rural farm or a non-hospital remote healthcare site such as a nursing post, this was calculated from the time of initial contact with the retrieval service.

Availability of mortality data was dependent on compliance with data entry into the database from individual institutions. Hospital databases were later accessed by the research group to determine missing 30-day and 120-day mortality for the three groups. The 30-day and 120-day mortality rate follow-up was 88% and 78%, respectively. Only patients with recorded mortality data were included in the mortality analysis.

The impact of presenting location on time to surgery, length of stay and 30-day and 120-day mortality was analysed. A two-tailed student’s t-test was used for comparison of means and Chi-squared test for independence of categorical data (percentage of patients achieving time to surgery). A post-hoc power analysis was performed using G*power [20] to detect a difference of 5 hours between the metropolitan and either the rural or outer metropolitan groups with an alpha of 0.05 which gave a power of 100% for both comparisons. All data was collated into a spreadsheet (Microsoft Excel 2011 for Mac, Microsoft Corporation, Redmond, WA, USA). Statistical analysis was carried out using IBM Corp SPSS (IBM Corp., Released 2019, IBM SPSS Statistics for Windows, Version 26.0, Armonk, NY, USA).

Ethical approval was granted by the Fiona Stanley Fremantle Hospital Group Quality Improvement Activity Program, approval no. 26196. This research was conducted in accordance with the Declaration of Helsinki and National Institute of Health (NIH) Guidelines.

## Results

There were 4477 patients (79%) in the metropolitan group, 910 (16%) in the outer metropolitan group and 304 (5%) in the rural group. The rural patients were younger and more likely to be of Aboriginal and/or Torres Strait Islander ethnicity compared to the metropolitan and outer metropolitan groups as demonstrated in Table 1.

**Table 1:** Mean age and ethnicity by region

Region	Metropolitan	Outer-metropolitan	Rural	Total
Total number of patients	4477	910	304	5691
Mean age (years)	82.6 (SD 9.9)	80.8 (SD 10.1)	76.1 (SD 12.4)	82.0 (SD 10.2)
Aboriginal and/or Torres Strait Islander	0.5%	0.7%	22.1%	1.6%

There was a statistically significant difference in gender proportions between the three groups (p<0.001), 72% of metropolitan patients were female, 68% of outer metropolitan patients were female and 60% of rural patients were female.

**Figure 1:**
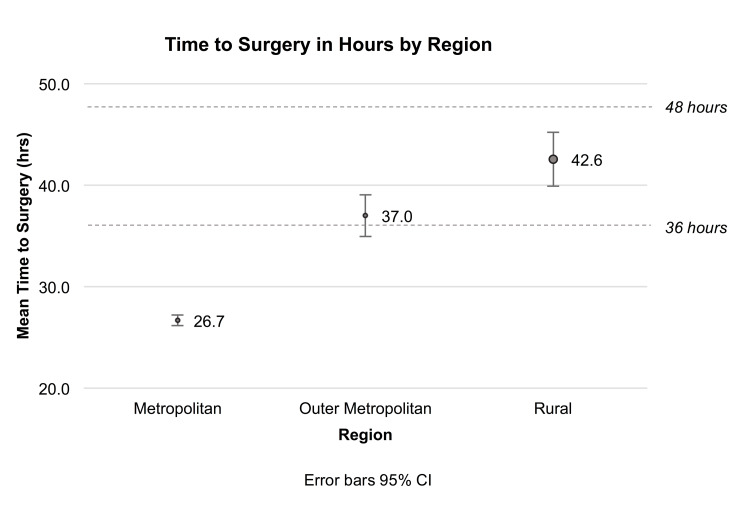
Time to surgery in hours by region with 95% Confidence Interval The dashed line demonstrates the target of surgery within 36 and 48 hours.

Time to surgery progressively increased with distance from the centre of the definitive surgery, as seen in Figure 1 (p<0.001).

**Figure 2:**
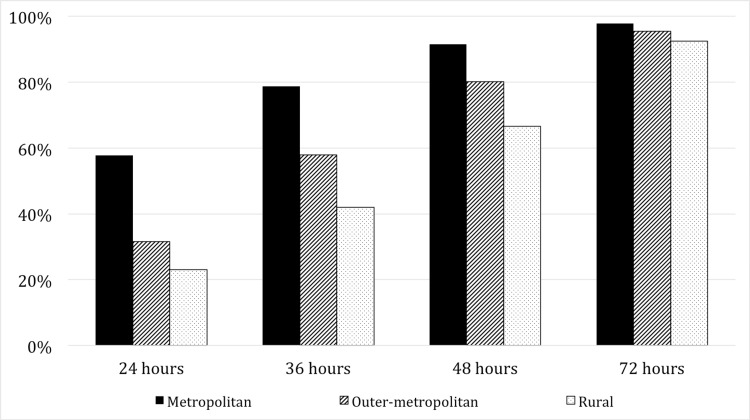
Percentage of each region group that reaches surgery, by hour targets

Outer metropolitan patients, despite being less than two and a half hours by road from a tertiary centre, had much lower rates of achieving surgery by the 36-hour (58.0% vs 78.7%, p<0.001) and 48-hour marks (80.2% vs 91.5%, p<0.001) when compared to their metropolitan counterparts. This was also true for rural patients at both the 36-hour and 48-hour time points compared to metropolitan patients (42.0% and 66.8% respectively, p<0.001). By the 72-hour mark, all patients had comparable rates of surgery. These trends over hour targets are demonstrated in Figure 2.

Mean acute length of stay was longer for rural patients (7.2 days), compared to outer-metropolitan (5.8 days) and metropolitan patients (5.5 days) (p<0.001).

There was 30-day mortality data for 3951 (88%) of the metropolitan patients, 801 (88%) of the outer metropolitan patients and 263 (86%) of the rural patients. There was 120-day mortality data for 3541 (79%) of the metropolitan patients, 693 (76%) of the outer metropolitan patients and 233 (76%) of the rural patients. Only patients with mortality data were included in the mortality analysis. No statistically significant difference was found in the 30- or 120-day mortality for outer-metropolitan (p=0.59 and p=0.37) or rural patients compared to metropolitan patients (p=0.41 and p=0.34, respectively). Although no difference in mortality was found between groups despite the statistically significant difference in time to surgery between groups, the whole cohort demonstrated a correlation between time to surgery and 120-day mortality as demonstrated in Table 2. The overall 30-day and 120-day mortality was 8.7% and 17.3%, respectively.

**Table 2:** Comparing 120-day mortality with various time to surgery targets achievement

Target	120-day mortality, reached target	120-day mortality, did not reach target	p-value
24 hours	16.1%	18.5%	0.02
36 hours	16.5%	19.5%	0.012
48 hours	16.8%	21.2%	0.006
72 hours	17%	28.7%	<0.001

## Discussion

Overall, despite large differences between patient groups, the time to surgery for hip fracture patients compares well to other hospitals in Australia with an average time to surgery in this study of 29 hours for all patients. We can see that on average, metropolitan and outer-metropolitan patient groups are achieving the desired target of 48 hours from the first presentation. As expected, a significantly lower percentage of outer-metropolitan and rural patients are achieving these targets. This is not a problem unique to WA. The study from New South Wales showed increased time to surgery for transferred patients [16]. However, it is worth noting that the study site Bega is a regional hospital, not a tertiary centre, and the time to surgery for all patients was longer than that in WA. WA tertiary hospitals all have dedicated trauma theatre lists and orthogeriatric services working in partnership with the orthopaedic teams. New South Wales’ overall state-wide time to surgery was comparable to our rural time to surgery but poorer than our metropolitan and outer metropolitan time to surgery [17].

The Australian & New Zealand Hip Fracture Registry annual reports calculate time to surgery from hospital of first presentation until the commencement of surgery [11-15] which is the same methodology of this paper. Compared to the overall ANZHFR time to surgery data in 2018-20 [13-15], both Western Australian patients who present directly to the operative hospital or who are transferred are getting to surgery faster than most places in Australia. Perhaps this represents a successful unified approach in WA across various services in prioritising hip fracture transfers and operative care.

Ontario, Canada, was used as a comparison to this study due to its large geographic size and similar logistical barriers to WA. Compared to their time to surgery of 93 hours for transferred patients [18], WA’s average 46.2 hours time to surgery for rural patients and 37 hours for outer-metropolitan patients is closer to recommended targets. This demonstrates reasonable results in WA despite logistical and geographical barriers to accessing timely surgery. Subsequent to this Canadian study, a new protocol in Ontario was created to facilitate increased priority of transfer with the goal of surgery within 48 hours [18].

Despite demonstrating a significant difference in time to surgery between the three patient groups, this study did not show a difference in 30- and 120-day mortality between groups. Harvey et al. reviewed New South Wales’ hip fracture patients over an eight-year period. They also reported a longer time to surgery for transferred patients, but no significant impact on 30-day mortality [21]. As far as the researchers are aware, these are the first two studies to demonstrate no increase in mortality despite transfer status and increased time to surgery. This suggests that there are other factors at play that need to be looked at closely.

There are a number of times to surgery barriers for outer-metropolitan and rural patients. Geographical location is obvious, although distance is not proportional to time delay. Rural patients requiring air transfer are transferred via the Rural Flying Doctor Service (RFDS). Limitations of aircraft availability, pilot hours, weather and other patient transfers can impair rural patients’ access to timely retrieval. Given the complexity, transfer will occur when an aircraft and crew become available, rather than when there is a bed on the other end. The relatively low time to surgery for rural patients despite the vast distances involved may reflect the partnership that exists between RFDS and metropolitan centers. Both parties understand how the other works and the standard of care expected. RFDS can provide nerve blocks prior to transfer and do prioritise hip fracture patients [22]. An RFDS transfer has the added benefit of 1:1 specialist care during transfer, which may impact patient outcomes and perhaps explain the lack of difference in mortality rates for the rural group despite increased time to surgery compared to the metropolitan patients.

For outer metropolitan patients, road transfer is a comparatively easier task, so delays are often the result of bed management and bed availability at the receiving tertiary centre, whereby the transfer will be delayed until bed access allows. This study showed that outer metropolitan patients are experiencing delays longer than what is accounted for by transfer alone, and this is an area for improvement. Ambulance bypass of outer metropolitan centres is an accepted strategy for other conditions such as major trauma, stroke and ST-elevation myocardial infarction, but it has also been employed for hip fracture in other parts of the world such as Ireland [23]. Clinical prioritisation of hip fracture patients by bed managers and administrative staff for earlier transfer may also reduce delays. These could be considered as strategies to reduce time to surgery for outer metropolitan centres.

A lack of access to an orthogeriatric and perioperative care team while waiting at an outer-metropolitan or rural site can also delay medical optimisation and result in a longer time to surgery. Outreach from tertiary centres to rural and outer-metropolitan hospitals to improve hip fracture pathways prior to transfer, including initiating peri-operative management and optimisation, completing appropriate comorbid screening, and liaison with theatre planning at the tertiary centre, may help to reduce delays to surgery once the patient has arrived in the operative location. A lack of experienced ward nurses in hip fracture care in a non-operative centre could also result in suboptimal treatment.

Strengths of this study include a broad data collection period of five years, combination with accurate mortality data and reliable times and dates of presentation and surgery as they are automatically recorded from hospital data. Surgery timing data was recorded for 97% of patients. The reason for delay to surgery was often not recorded so analysis of this was not possible. Patients were excluded due to unclear transport method, as this data point was not specifically recorded. Follow-up data including morbidity, and length of stay in rehabilitation beyond the acute admission was often missing so further conclusions about the impact of transfer time on long-term outcomes are limited, although this was not the primary aim of the study and the effects of delays to surgery are well established. Patients are manually entered into the QoCR database by a clinical nurse specialist with the specific role of recording this data, and because of this, it is unlikely that many hip fracture patients are missed.

Statistical analysis of patient factors did not account for covariates and this is a limitation of the study. Known risk factors for delay in hip fracture surgery are comorbidity score, race, insurance status, hospital region and day of admission [24]. All hip fracture patients in this study were treated at a public hospital, so insurance status is not relevant. Comorbidity score and race were not available for all patients. Although the day of the week was not analysed, with over 5000 patients in the study there is not expected to be a difference in the day of the week of presentation between the three groups. The only remaining source of bias was the patient location, or hospital region, and analysing this was the primary aim of this paper.

Further research should focus on analysing specific reasons for delay with a view to improving transfer pathways. This would help to guide direct interventions to improve access for our rural and outer metropolitan patients. Of note, 22% of our study’s rural patient group were Indigenous and/or Torres Strait Islander, compared to less than one percent of the metropolitan and outer-metropolitan groups. This may have influenced time to surgery for this patient group due to patient factors other than location. Further analysis could examine if patient factors such as ethnicity affect time to surgery or are contributing to disparities in hip fracture care in Western Australia.

## Conclusions

Reduced time to surgery is a known, well-studied target for hip fracture patients. Patients presenting outside of the metropolitan area are subject to an average delay to surgery of 11 hours for outer metropolitan areas and 16 hours for rural areas compared to patients who present directly to an operative hospital. Delay for outer-metropolitan patients is disproportionately longer than transit time alone and may provide scope for improvement. Future research should focus on the specific reasons to delay and improving transfer processes, particularly for outer-metropolitan patients with this disproportionately longer delay. Overall, despite servicing a large area and needing to overcome large distances, time to surgery and mortality for hip fracture patients in Western Australia is in keeping with accepted standards.
